# Investigation of Droplet Spreading and Rebound Dynamics on Superhydrophobic Surfaces Using Machine Learning

**DOI:** 10.3390/biomimetics10060357

**Published:** 2025-06-01

**Authors:** Samo Jereb, Jure Berce, Robert Lovšin, Matevž Zupančič, Matic Može, Iztok Golobič

**Affiliations:** Faculty of Mechanical Engineering, University of Ljubljana, Aškerčeva cesta 6, SI-1000 Ljubljana, Slovenia; samo.jereb@fs.uni-lj.si (S.J.); jure.berce@fs.uni-lj.si (J.B.); matevz.zupancic@fs.uni-lj.si (M.Z.); iztok.golobic@fs.uni-lj.si (I.G.)

**Keywords:** droplet impact, superhydrophobic surface, machine learning, maximum spreading coefficient, droplet rebound, rebound efficiency, laser-textured surface

## Abstract

The spreading and rebound of impacting droplets on superhydrophobic interfaces is a complex phenomenon governed by the interconnected contributions of surface, fluid and environmental factors. In this work, we employed a collection of 1498 water–glycerin droplet impact experiments on monolayer-functionalized laser-structured aluminum samples to train, validate and optimize a machine learning regression model. To elucidate the role of each influential parameter, we analyzed the model-predicted individual parameter contributions on key descriptors of the phenomenon, such as contact time, maximum spreading coefficient and rebound efficiency. Our results confirm the dominant contribution of droplet impact velocity while highlighting that the droplet spreading phase appears to be independent of surface microtopography, i.e., the depth and width of laser-made features. Interestingly, once the rebound transitions to the retraction stage, the importance of the unwetted area fraction is heightened, manifesting in higher rebound efficiency on samples with smaller distances between laser-fabricated microchannels. Finally, we exploited the trained models to develop empirical correlations for predicting the maximum spreading coefficient and rebound efficiency, both of which strongly outperform the currently published models. This work can aid future studies that aim to bridge the gap between the observed macroscale surface-droplet interactions and the microscale properties of the interface or the thermophysical properties of the fluid.

## 1. Introduction

Superhydrophobic surfaces, often inspired by natural examples like the lotus leaf, represent a class of surfaces characterized by their extreme water repellency [1,2]. These surfaces are defined by a high apparent water contact angle (typically greater than 150°) and low contact angle hysteresis (below 10°), which ensures minimal resistance to the movement of water droplets, resulting in low roll-off and sliding angles. The key to achieving surface superhydrophobicity lies in a combination of surface chemistry and micro-nanoscale roughness, creating air pockets that reduce the contact area between the surface and water. Superhydrophobic surfaces have a range of practical applications across various fields. In environmental science, they enable water harvesting technologies, mimicking the mechanisms of desert beetles and spider silk to collect and channel water efficiently [3,4,5]. Their self-cleaning capabilities, inspired by, for example, the lotus leaf, make them invaluable in coatings for glass, textiles and solar panels, where cleanliness is essential for performance and efficiency [6]. Additionally, superhydrophobic materials are increasingly being explored in anti-icing applications [7,8], drag reduction for ships and pipelines [9], liquid separation and biomedical devices [10,11]. By studying and mimicking natural examples of superhydrophobic surfaces, novel surface treatments can be developed to surpass the current state-of-the-art superhydrophobic surfaces.

Extreme non-wetting properties induce particular surface interactions with liquid droplets upon impact, which are subject to several parameters. When impacting a surface, the droplet experiences a rapid interplay of inertial, viscous and surface tension forces, leading to several possible outcomes, including complete rebound, partial rebound, splashing (fragmentation) or deposition [12]. The specific behavior depends on the droplet’s properties, impact velocity and surface characteristics. These interactions are often analyzed using dimensionless numbers, i.e., Weber number (*We*), which represents the ratio of inertial to surface tension forces, and the Reynolds number (*Re*), which describes the ratio of inertial forces relative to viscous forces.

(1) $$We=\frac{\rho u_{0}^{2}D_{0}}{\sigma }$$

(2) $$Re=\frac{\rho u_{0}D_{0}}{\mu }$$

The formulations include liquid density *ρ*, droplet impact velocity *u*_0_, droplet diameter *D*_0_, surface tension *σ* and dynamic viscosity *μ*.

At low *Re* and *We* values, the droplet is expected to spread minimally upon impact and rebound cleanly with minimal deformation, resulting in a high restitution coefficient due to limited energy dissipation [13]. In this regime, the maximum spreading factor *β*_max_ remains relatively low. As *Re* and *We* increase, inertia begins to play a more significant role, leading to greater droplet deformation and increased spreading as the droplet flattens extensively before rebounding [14]. At intermediate *Re* and *We* values, droplets typically achieve a clean rebound on superhydrophobic surfaces, minimizing pinning and energy loss. At higher *We* values, the droplet’s inertia overcomes surface tension, potentially causing splashing, where satellite droplets are ejected radially during impact [15]. In this regime, rebound efficiency decreases due to significant energy loss from droplet fragmentation and viscous dissipation. When *Re* is high, but *We* remains moderate, a phenomenon known as partial rebound may occur, where the droplet detaches but leaves behind small residues due to localized pinning. This outcome is detrimental in applications requiring a complete rebound, such as anti-icing or self-cleaning surfaces. Although Reynolds and Weber numbers are widely used to characterize droplet impacts, merging multiple physical effects into a single parameter may obscure the influence of individual contributing factors.

Lately, machine learning (ML) has emerged as a powerful tool for modeling various processes, including the spreading and rebound behavior of droplets, as it offers the ability to capture complex, nonlinear relationships that are difficult to address with traditional models. Similar to experimental analysis, studies that reported the use of data-driven methods most often adopt dimensionless numbers as inputs to a given model while focusing primarily on droplet spreading. In this way, Heidari et al. [16] optimized a nonlinear auto-regressive exogenous–artificial neural network (NARX-ANN) to achieve an R^2^ of 0.993 when predicting droplet spreading within a group of 1220 datapoints from the literature. Instead, Tembely et al. [17] used linear regression and decision tree model, as well as two ensembles, i.e., random forest (RF) and gradient boosted regression tree (GBRT), to predict droplet spreading on literature-reported data of surfaces with differing wettability. The best-performing GBRT model (R^2^ = 0.963) highlighted the importance of *Re* and *We* numbers compared to the third predictor, the apparent contact angle. Pierzyna et al. [18] proposed a data-driven model for predicting the deposition-splashing threshold on dry, smooth surfaces, leveraging support vector classification while incorporating experimental uncertainties. Azimi Yancheshme et al. [19] recorded water droplet impacts on hydrophobic and superhydrophobic surfaces, using the results to train an RF model for the classification of five distinct outcomes—deposition, partial bouncing, full bouncing, bouncing–splash transition and splash. With a classification accuracy of 98%, the trained model predicted splashing at *We* numbers over 100–120, regardless of the superhydrophobic surface or impacting droplet characteristics, while only deposition was observed on hydrophobic surfaces. The same group [20] also showed that the droplet spreading factor is independent of surface characteristics and *We* number at contact angles over 150°. Adopting an RF model, they concluded that at *We* < 60 and CA < 150°, the increased contact angle leads to a decrease in the spreading factor. Instead, for *We* > 60–80 and CA < 150°, the latter is proportional to the spreading factor. Apart from applying ML to droplet rebound at room temperature, several studies extended the conditions to different temperature levels. Keshavarzi et al. [21] applied logistic regression, decision tree and random forest models to the classification of droplet rebound at temperatures of −20 °C, −10 °C and 25 °C. All three models highlighted the dominant contribution of the *Re* number and surface temperature on the observed impact regime, while the RF model also assigned high importance to the *We* number. Instead, the surface properties, i.e., wetting and roughness, were deemed less influential by all models. The classification of impacting droplet interactions was also studied by Au-Yeung et al. [22], using an artificial neural network to construct a phase diagram of impact outcomes on a nanostructured heated surface (25–550 °C), distinguishing between nanopillar geometric parameters. The group also adopted support vector regression to predict the spreading factor, revealing a universal value of ~ 3.84 for high *We* numbers (≥2000), where the maximum spreading becomes independent of surface properties. Yang et al. [23] reported the use of a back propagation neural network model for the prediction of the spreading factor, as well as contact time on a supercooled surface (Δ*T* = 10–35 K). The authors then attempted to classify icing patterns using a convolutional neural network, achieving up to 90.5% accuracy. In addition to the aforementioned research, more specific use cases of machine learning have been reported for droplet impacts, with authors showing encoder–decoder image generation of splashing droplet morphologies at different impact velocities [24], while others used an ensemble model to predict the droplet impact force on a curved surface [25].

Despite the rather limited number of available publications, much work has already been invested in incorporating machine learning into the analysis of droplet impact dynamics, showcasing the possibility of revealing the underlying physical phenomena. However, while a few studies addressed the spreading phase, the subsequent receding phase and rebound have not been properly analyzed with data-driven methods. The latter is critical to elucidate the influence of individual parameters on all stages of droplet rebound, which can serve as the foundation for fabricating advanced surfaces to achieve a targeted surface–droplet interaction in given conditions. In addition, several previous studies have highlighted the possible influence of fluid properties, which remain poorly explored. This is detrimental to extending the range of applicability of a given superhydrophobic surface, where the effect of changes in fluid thermophysical properties might influence the expected impact outcome.

To address these critical gaps in knowledge, we present a novel use of data-driven isotropic exponential Gaussian process regression to model a large experimental data cluster of droplet impacts on superhydrophobic aluminum. We consider the most common droplet-related parameters (diameter and impact velocity) and surface topography while also modeling the influence of fluid properties (density, dynamic viscosity and surface tension) of five water–glycerin binary mixtures. Importantly, we analyze the individual influence of aforementioned parameters on three characteristic metrics—contact time, maximum spreading coefficient and rebound efficiency, covering all stages of droplet impact. Finally, we develop empirical correlations for predicting the maximum spreading coefficient and rebound efficiency, both of which strongly outperform all of the currently published models.

## 2. Materials and Methods

The following paragraphs briefly describe the procedure of superhydrophobic surface preparation, experimental setup and measurement protocol and data reduction, which were used to acquire the data on droplet impact used later on for machine learning. A more detailed description of the applied methodology and the publicly available experimental dataset can be found in Ref. [26].

**Figure 1:**
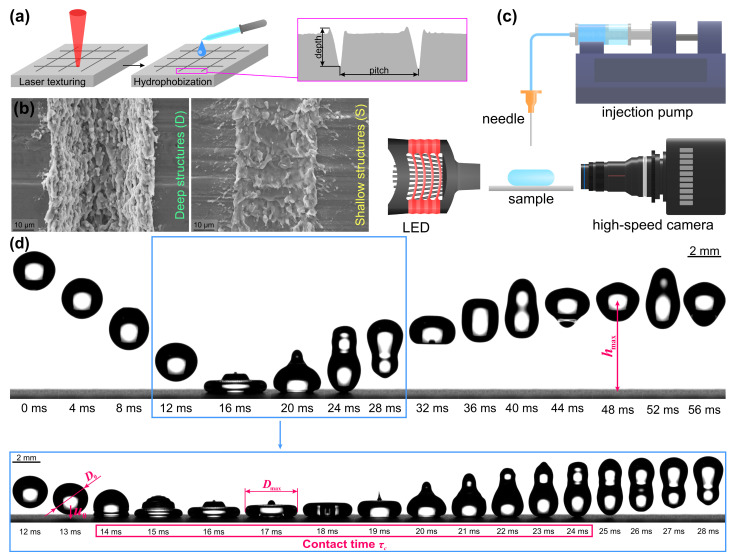
Methodology for droplet impact data acquisition: (**a**) preparation of superhydrophobic surfaces, (**b**) SEM images of laser-textured microchannels with depths of 25 (D) and 6 μm (S), (**c**) experimental setup for droplet impact measurements and (**d**) image sequence of droplet rebound illustrating key impact parameters.

Superhydrophobic surfaces were fabricated on aluminum alloy plates (1050A H24, ≥ 99.5% Al) via a two-step preparation process combining laser texturing and hydrophobization, as summarized in Figure 1a. Laser texturing was performed using a nanosecond fiber laser system (FL Mark-C with JPT Opto-electronics “M7 30 W” MOPA source), scanning the laser beam in a crosshatch pattern with a center-to-center distance of 50, 100, 200, 400, 600 and 800 μm between successive passes. Two different sets of laser-texturing parameters were used to fabricate microchannels with a depth of either ~6 or ~25 μm. Scanning electron microscopy (SEM) images of the microchannels are shown in Figure 1b. After texturing, the surfaces were hydrophobized by applying several drops of 3 mM solution of 1H,1H’,2H,2H’-perfluorododecil-1-phosphonic acid (FDPA) in 2-propanol to each sample, forming a self-assembled monolayer. After treatment, the surfaces exhibited exceptional water-repellency with an apparent contact angle of water droplets above 160°. A more detailed evaluation of surface wettability is provided in the Supplementary Material Table S1.

Droplet impact measurements were conducted on a custom-made experimental setup, schematically depicted in Figure 1c, using water–glycerin binary mixtures with 0, 20, 60, 78 and 91 wt.% glycerin. A syringe pump and a blunt-tip needle were used to produce droplets of controlled size, which were released onto the samples from five different heights to vary the impact velocity. Droplet impact dynamics were captured using a high-speed optical camera, with a white LED providing back-side illumination. The liquid temperature was recorded prior to each measurement to determine its thermophysical properties.

The captured footage of droplet rebounds was processed using a custom-developed image-processing algorithm based on contour detection to extract the key parameters describing the droplet–surface interaction for each measurement, including (i) initial droplet diameter prior to impact *D*_0_, (ii) droplet impact velocity *u*_0_, (iii) droplet–surface contact time *τ*, (iv) maximum droplet radial extension *D*_max_ and (v) droplet rebound height *h*. The aforementioned parameters are visually presented in an image sequence of a representative droplet rebound recording in Figure 1d. The initial droplet diameters and impact velocities were used to obtain the non-dimensional Weber and Reynolds numbers, characterizing the properties of the droplets prior to impact, while the maximum spreading coefficient (*β*_max_ = *D*_max_*/D*_0_) and energy efficiency of the rebound *η* were calculated to quantify the outcome of the droplet rebound. The latter was calculated as the ratio between the droplet’s potential energy as it reached the highest point after the rebound and its maximum kinetic energy prior to impacting the surface:

(3) $$\eta =\frac{E_{potential}}{E_{kinetic}}$$

### 2.1. Data Preparation

**Figure 2:**
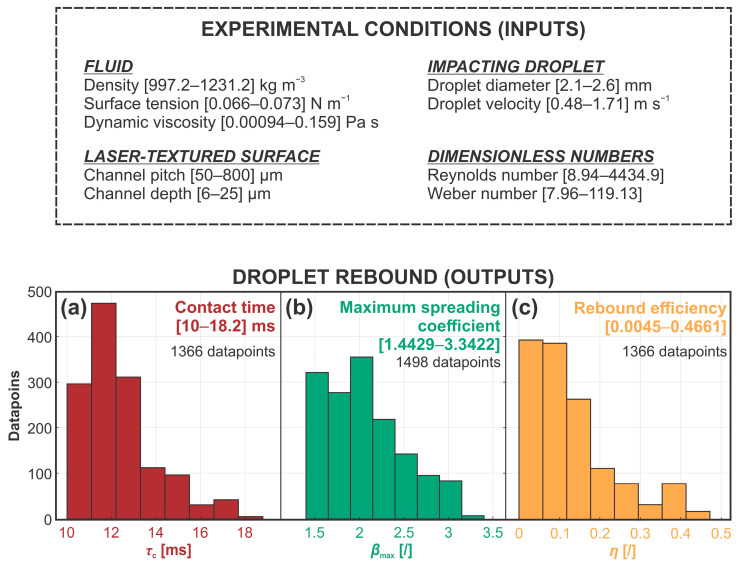
Data structure and range of predictor (inputs) and prediction (outputs) parameters: (**a**) contact time, (**b**) maximum spreading coefficient and (**c**) rebound efficiency.

The results of 1498 droplet impact experiments at various conditions were first structured into an array of predictors (inputs) and predictions (outputs). The latter group included contact time, maximum spreading coefficient and rebound efficiency. To separately predict each of the three parameters, the same set of seven predictors was chosen, consisting of droplet diameter, droplet velocity, fluid density, fluid viscosity, fluid surface tension, as well as microchannel pitch and depth. Note that in some tested experimental conditions, a definitive droplet rebound was not observed, meaning that the contact time and rebound efficiency were not available for 132 datapoints. As a result, the size of the data array for these two outputs was reduced to 1366 as opposed to the full array of 1498 datapoints that were used to predict the spreading coefficient. The data structure is shown in Figure 2, with the parameter histograms showing the range and distribution of each parameter to be predicted.

**Figure 3:**
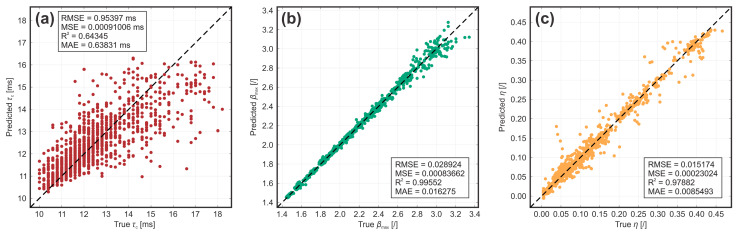
Predictive performance metrics of the optimized IE GPR model for (**a**) contact time, (**b**) maximum spreading coefficient and (**c**) rebound efficiency.

For each predictor–prediction combination, the aim of this work was to train a multi-input single-output (MISO) machine learning model capable of accurate predictions within our experimental data range:

(4) $$\left\{\tau_{c}\;,\;\beta_{max},\;\eta \right\}=M\left(D_{drop}\;,v_{drop}\;,\rho \;,\mu \;,\sigma \;,pitch\;,depth\right)$$

totaling three trained and validated machine learning models. To avoid possible influences of different model architectures, we used a single ML archetype, chosen from 28 available models inside the MathWorks MATLAB R2024b Regression Learner Toolbox. Firstly, each input parameter was normalized with standard Z-test normalization:

(5) $$z=\frac{\left(x-\bar{x}\right)}{\sigma }$$

where *x* is a single value, $\bar{x}$ is the mean and σ the standard deviation of the sample population. This procedure preserves the properties of the initial distribution while removing possible bias towards larger predictor values during subsequent model training. Afterwards, normalized data with an 80-to-20 training–testing data split were used to train, validate and test the 28 models with default hyperparameter values for each of the three output parameters. The result of training (in the form of 5-fold cross-validation prediction metrics) and testing (prediction metrics on the unseen data) of each model is shown in the Supplementary Material Tables S2–S4. One can see that the isotropic exponential Gaussian process regression (IE GPR) model achieved the best collective performance, warranting its selection for further optimization. Thus, the IE GPR hyperparameters were fine-tuned through 100 iterations of Bayesian optimization on the whole dataset, resulting finally in three optimized MISO models with predictive performance metrics shown in Figure 3.

Note that, even after optimization, the prediction of contact time is much less reliable compared to the other two parameters, which is due to very small differences in values even at different conditions, meaning that experimental uncertainties play a larger role in the measured outcome of droplet rebound.

## 3. Results and Discussion

### 3.1. Contact Time

Once a machine learning model is trained, Shapley values [27] may be computed to interpret the contribution of individual predictors in a single prediction query point. If this is done for all datapoints and averaged, the mean importance of each predictor on model output is obtained. For the contact time ML model, this analysis highlights the importance of microchannel pitch in comparison to other parameters. Surprisingly, the model showed little to no correlation between the contact time and the droplet diameter, liquid density and dynamic viscosity, despite their well-established relationship *τ*_c_ ~ (*ρr*_0_^3^/*γ*)^1/2^ [28]. It is worth noting that due to the relatively poor model fit described above, the Shapley ratios for contact time should not be trusted implicitly, and a larger scatter of experimental data should be employed to draw definitive conclusions regarding parameters with lesser importance.

**Figure 4:**
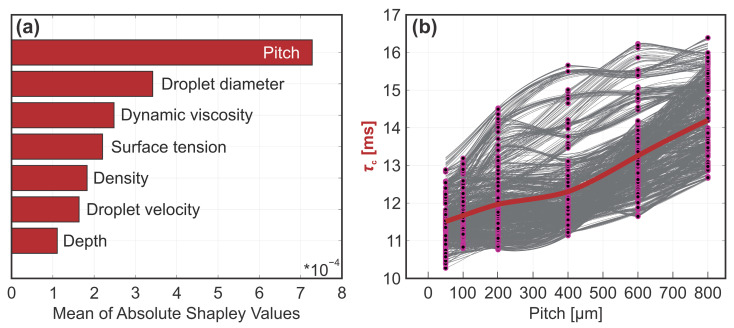
Analysis of contact time: (**a**) individual predictor importance and (**b**) ICE plot showing the influence of pitch.

The complexity of most trained ML models hinders their interpretation if one wishes to correlate a predictor value to the change in the output value. One way to achieve this is with individual conditional expectation (ICE) plots [29], visualizing the dependence of the prediction variable on individual features. Each line in an ICE plot represents one of *N* data samples, where the feature of interest is varied within a grid while the rest of the features are kept constant, and the influence of this variation on the output is plotted. The averaged dependence is represented by the bold line, i.e., the partial dependence plot (PDP), while each of the *N* observations is plotted as a point on top of the corresponding line. If only the PDP is used for model interpretation, possible heterogenous dependences can be overlooked. For the optimized IE GPR model for contact time prediction, we generated the ICE plots for the most influential predictor (i.e., microchannel pitch) with all 1366 datapoints available (see Figure 4b). The PDP plot shows a progressive trend, indicating that an increase in the pitch value prolongs the contact time. This is consistent with expectations, given that sparser channels increase liquid adhesion, which, in turn, means that the droplet rebounds slower, if at all. All ICE plots show non-diverging behavior, signifying that no heterogenous dependency is present in the model and that in the whole data range, the increase in pitch causes an increase in contact time.

### 3.2. Maximum Spreading Coefficient

Next, we focus on the maximum spreading coefficient, signifying the maximum horizontal extension of the droplet during the impact with respect to the initial droplet diameter. Although the maximum spreading coefficient accounts only for the spreading stage of droplet rebound, it is likely the most extensively researched rebound descriptor, with numerous physical and empirical models correlating its value to the droplet impact characteristics, liquid thermophysical properties and surface wettability [30,31,32,33,34,35,36,37,38]. However, most proposed correlations employ non-dimensional *We*, *Re* and *Oh* numbers combining multiple parameters into a single value to reduce the complexity of modeling, potentially obscuring the influence of individual contributions. Contrary to physical and empirical modeling, data-driven modeling is not constrained by the complexity of the phenomenon, allowing us to identify the influence of each individual contributing parameter independently.

**Figure 5:**
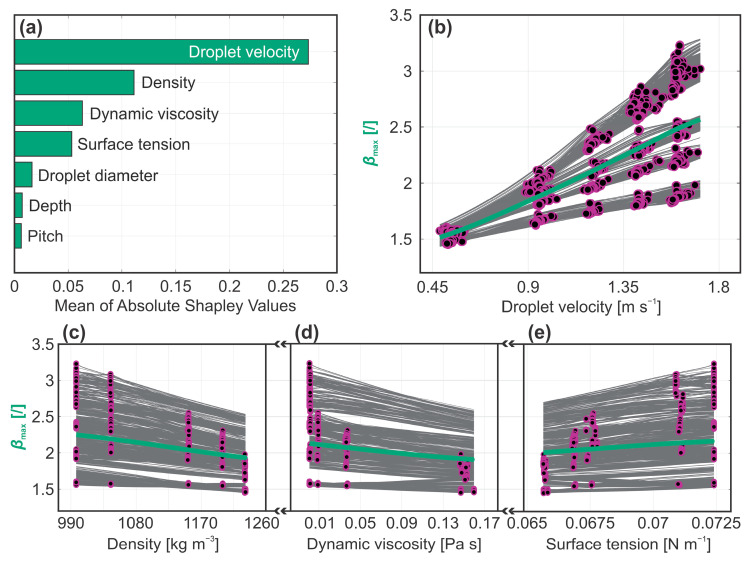
Analysis of maximum spreading coefficient: (**a**) individual predictor importance and (**b**–**e**) ICE plots for the most influential predictors.

Based on the calculated Shapley values shown in Figure 5a, droplet spreading is primarily affected by impact velocity, followed by the thermophysical properties of the liquid. Interestingly, neither the depth nor pitch of the microchannels show any considerable effect on the spreading of the droplet. The latter suggests that the surface topography does not directly influence the droplet dynamics during the spreading stage of the rebound. Previous research has shown that the spreading behavior of a droplet can be significantly altered when the surface features are on a scale comparable to that of the droplet [39,40,41,42]; however, this is not the case in the present study. Note that the scale of the microchannels is orders of magnitude smaller than the size of the droplet, supporting our conclusions. Nevertheless, the surface microstructure is inherently linked with surface wettability, which has been previously identified as a key factor in droplet spreading dynamics [43]. In our case, all the tested surfaces exhibited similar wetting properties regardless of the microchannel pitch and depth, which may explain the observed weak correlation between the maximum spreading coefficient and surface characteristics.

To correlate a predictor value change to the change in the value of the prediction, we computed the ICE plots for the most influential predictors identified from the Shapley values, as we did for the contact time. Figure 5b–e presents the individual influence of droplet velocity, liquid density, dynamic viscosity and surface tension. The PDP plot for droplet velocity shows a progressive increase, indicating that the droplet spreads further when impacting the surface at higher velocities. This aligns with expectations, as greater impact velocity corresponds to higher kinetic energy, which is converted into surface energy upon impact, leading to increased spread. In contrast, the PDP plots for both density and dynamic viscosity exhibit a falling rate trend, suggesting that droplet spreading decreases as the value of the two thermophysical properties increases. While the correlation between higher dynamic viscosity and reduced spreading is rather trivial since viscosity inherently represents the fluid’s resistance towards shearing deformation, the same cannot be said for liquid density. One could argue that increasing the liquid density should result in higher kinetic energy and thereby enhance spreading rather than hindering it. This deviation from expectation may stem from the inherent link between the density and dynamic viscosity of the liquids used to acquire the experimental dataset. A similar issue arises with surface tension, where the ICE plots show a positive slope, suggesting that higher surface tension leads to increased droplet spreading. Since surface tension opposes the spreading of a liquid, an increase in its value should reduce the maximum spreading coefficient rather than enhance it. Note that in the ML training dataset, variations in liquid density, dynamic viscosity and surface tension were achieved by altering the composition of a water–glycerin binary mixture. While this approach provides data points at different values of the three thermophysical properties, the parameters are inherently co-dependent, which obscures the influence of the individual contributor. When examining the maximum spreading coefficient as a function of the water–glycerin composition, a negative trend is observed with increasing glycerin content for all three parameters. Based on previous research, reduced spreading at higher glycerin fraction is dominated by the change in dynamic viscosity, indicating that the importance of liquid density and surface tension within our model is likely overestimated [44]. Nonetheless, the model is able to accurately predict the maximum spreading coefficient for a given composition of water–glycerin mixture. To generalize the model and make it universally applicable to any liquid, the dataset used for training should incorporate binary mixtures of other liquids to mitigate the interdependence among thermophysical properties.

### 3.3. Rebound Efficiency

Lastly, we consider the rebound efficiency, comparing the energy retained by the droplet after the rebound to its initial energy prior to impact. In the literature, this parameter is often expressed in its squared form as the restitution coefficient ($\varepsilon =\sqrt{\eta }$), which compares the droplet’s velocity after the rebound to its velocity before impact. Regardless of the form, it is arguably the most comprehensive descriptor of droplet rebound dynamics among the three parameters used in this study. Its magnitude, or more specifically, its deviation from unity, depends on energy dissipation throughout the entire rebound process, encompassing droplet spreading and retraction, as well as the time during which the droplet is airborne.

**Figure 6:**
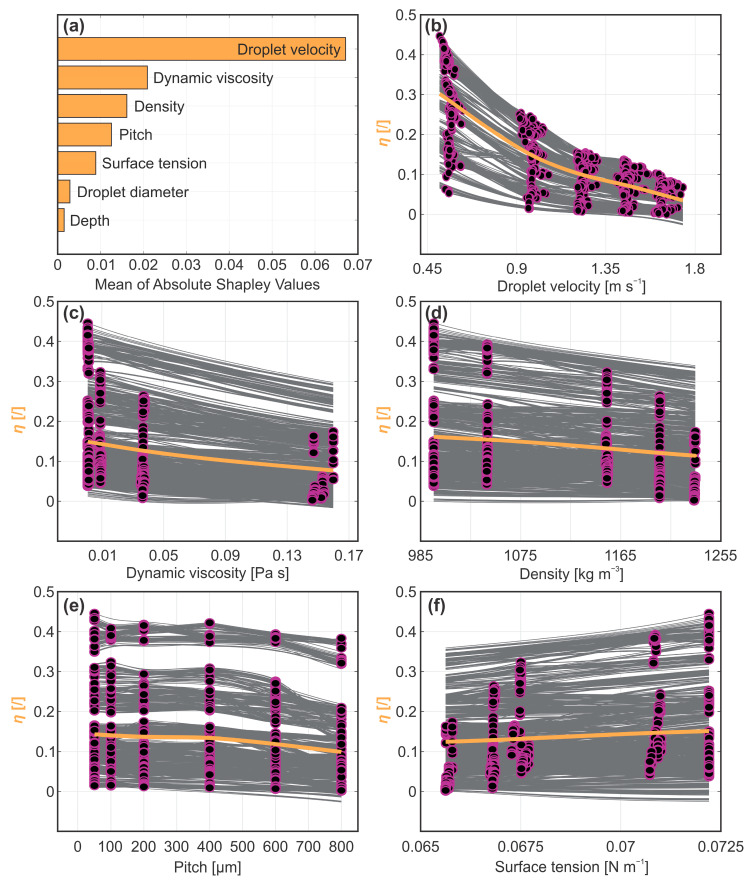
Analysis of rebound efficiency: (**a**) individual predictor importance and (**b**–**f**) ICE plots for the most influential predictors.

To identify the most influential parameters, we once again calculated the mean importance of individual predictors on the output parameter based on the computed Shapley values (Figure 6a). Similar to the maximum spreading coefficient, the analysis indicates that the rebound efficiency is primarily influenced by droplet velocity, followed by the liquid’s thermophysical properties. Since the contributions of individual liquid properties are likely obscured by their inherent interdependence and are dominated by the effect of dynamic viscosity (as discussed in Section 3.2), only the latter is considered in the following discussion. For a detailed analysis of the influence of droplet velocity and dynamic viscosity on the rebound efficiency, we now refer to the generated ICE plots shown in Figure 6b,c. The corresponding PDP plots for both predictor parameters exhibit a negative trend, indicating greater energy loss with increasing values. This energy loss is likely a result of viscous dissipation, with both parameters showcasing trends consistent with scaling observed within the function *ϕ*~*μ*(*u*_0_/*L*)^2^ [34].

Unlike droplet spreading, rebound efficiency also appears to be affected by microchannel pitch. This suggests that the pitch distance between microchannels affects the rebound dynamics only after the maximum spread is reached. Upon reaching its maximum horizontal extension, the droplet begins to contract into its equilibrium shape, driving the three-phase contact line into an inward motion. This retractive movement is opposed by adhesive forces between the liquid and the surface. As previously discussed, sparser microchannels increase the fraction of the non-textured surface area and thereby enhance liquid adhesion, resulting in greater energy dissipation at larger microchannel pitches [45]. The latter is evident from the observed falling rate trend in the PDP plot for the microchannel pitch shown in Figure 6e. Notably, the rebound efficiency showed no correlation with the microchannel depth, suggesting that the liquid does not penetrate deeply enough into the microchannel to reach the bottom.

### 3.4. Prediction with Dimensionless Numbers—Re, We

The Reynolds and Weber numbers were used to additionally test the predictive approach with derived non-dimensional parameters more commonly found in the literature models, yielding two more input–output combinations:

(6) $$\left\{\eta ,\beta_{max}\right\}=M\left(Re,\;We,\;pitch,\;depth\right)$$

Note that the prediction of contact time was not considered due to the already discussed poor predictive performance related to small experimental value scatter. For the other two parameters, the same model architecture was adopted (i.e., the IE GPR), which was optimized and validated in the same way as described previously in Section 2.1. The resulting predictive performance metrics for each output parameter are given in Table 1, where one can observe slightly lower values when compared to predictions with a full set of seven predictors.

**Figure 7:**
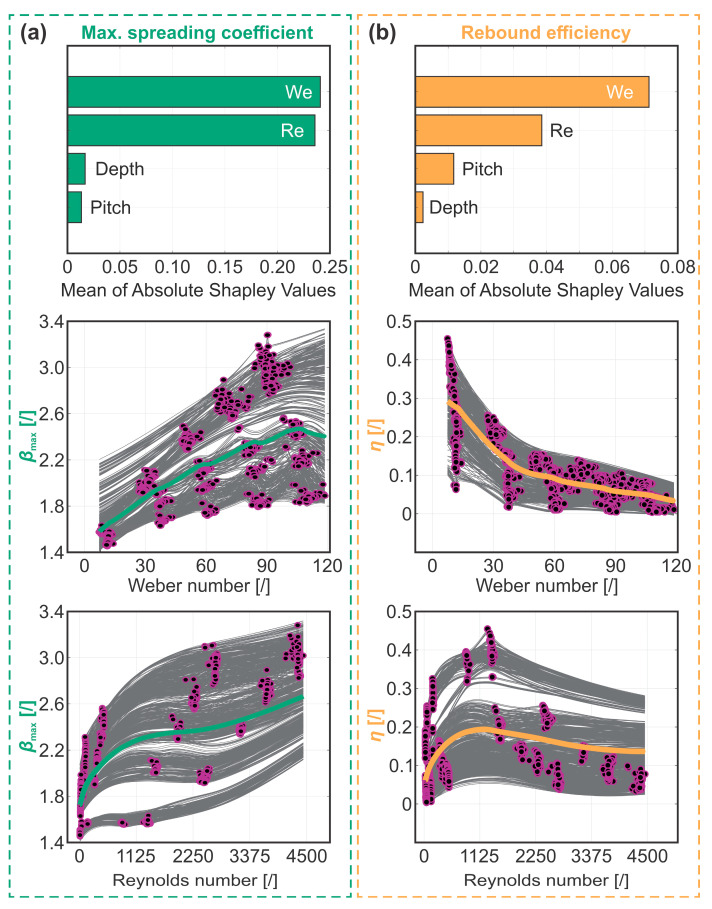
Analysis of (**a**) maximum spreading coefficient and (**b**) rebound efficiency with non-dimensional Reynolds and Weber numbers.

Focusing first on the analysis of the maximum spreading coefficient shown in Figure 7a, the model assigns comparable importance to the Weber and Reynolds numbers when making predictions while showing no correlation to surface morphology, consistent with the previous seven-predictor model. Since the droplet spreading behavior is predominantly governed by impact velocity and liquid viscosity, the Reynolds number, describing the ratio between the inertia and viscous forces, is presumably the more comprehensive descriptor of the phenomenon among the two dimensionless numbers. From the PDP plot relating the Reynolds number to the maximum spreading coefficient, we observe two distinct regions with different trends, separated by a Reynolds number of ~2000. At lower *Re* numbers, the spreading behavior is primarily dominated by the liquid’s viscosity, while impact velocity becomes the governing factor in the region of higher *Re* numbers. Around *Re* ≈ 2000, the influence of both parameters was minimal, as evidenced by the inflection of the PDP curve in this range. The Weber number, comparing the inertia to surface tension forces, was primarily altered through the variation of initial droplet height; therefore, a similar trend to that predicted by the model trained on the seven predictor parameters for the impact velocity is expected. While the PDP plot shows a continuous rise in the maximum spreading coefficient up to a Weber number of ~105, a gradual decline is observed as the Weber number increases further. The latter suggests that a Weber number of approximately 105 may be optimal for droplet spreading; however, this inflection point lacks a clear physical basis and is likely a result of non-uniform sampling of the measurements, as data at Weber numbers above 100 were obtained exclusively using high-viscosity mixtures, which inherently limit droplet spreading. If measurements across the full range of Weber numbers were performed using fluids with varying viscosities, this decline would likely not be observed.

Next, we analyze the rebound efficiency, as depicted in Figure 7b. The ML model, based on two non-dimensional parameters, identifies the Weber number as the most influential parameter in predicting the rebound efficiency, followed by the Reynolds number. Consistent with the seven-predictor model, the non-dimensional analysis also reveals a correlation with the microchannel pitch while showing no dependence on microchannel depth. The PDP curve relating the Weber number to the rebound efficiency reveals a negative trend, closely resembling the one for impact velocity that was produced by the model trained on seven predictors. Contrary to the maximum spreading coefficient, this trend appears unaffected by the non-uniformity of sampling at Weber numbers above 100 since both higher impact velocity and viscosity promote energy dissipation, thereby reducing rebound efficiency. The individual effects of the two parameters are more clearly discernible in the PDP curve for the Reynolds number, where we once again observe two distinct regions separated at *Re* ≈ 1125. Below this threshold, where viscous effects dominate, the rebound efficiency gradually rises as the Reynolds number increases, while in the region above 1125, where inertial effects become prominent, the rebound efficiency steadily decreases. The peak in rebound efficiency around *Re* ≈ 1125 suggests that an optimum for maximizing the droplet rebound exists in this value range. In contrast to the inflection point observed in the analysis of the maximum spreading coefficient, this local maximum in rebound efficiency is supported by a clear physical interpretation, as both viscous and inertial influences are minimized in this range.

The PDP curves relating the Reynolds and Weber number to the maximum spreading coefficient or rebound efficiency can also serve as a foundation for building an empirical correlation relating the dimensionless numbers to each rebound parameter. We attempted this by first populating the parameter space in order to obtain an equally spaced cloud of datapoints. To do this, a bivariate *Re*-*We* PDP plot was generated for *β*_max_ and *η* from their respective optimized IE GPR model. Both point clouds were generated with *We* numbers between 5 and 120 with a step of 1 and for *Re* numbers from 5 to 4500 with a step of 45, corresponding to the range of experimental values used in this study. In this way, a matrix of 11,600 equally spaced *Re*-*We* combinations with corresponding model-predicted values of *β*_max_ and *η* was obtained. The point cloud was then fed into a nonlinear least squares (NLLS) optimization algorithm, minimizing the summed square of the residuals while reducing the importance of outliers using bisquare weights. For each dimensionless number, a custom function was assumed based on the PDP plots shown in Figure 7 and fed into the NLLS optimization routine. It can be observed that *β*_max_ can be approximated by two multiplied exponentially increasing functions of *We* and *Re* numbers, yielding the following empirical correlation after optimization:

(7) $$\beta_{max}=1.1885+0.0397\cdot {We}^{\frac{\pi }{6}}\cdot {Re}^{\frac{\pi }{18}}\;$$

**Figure 8:**
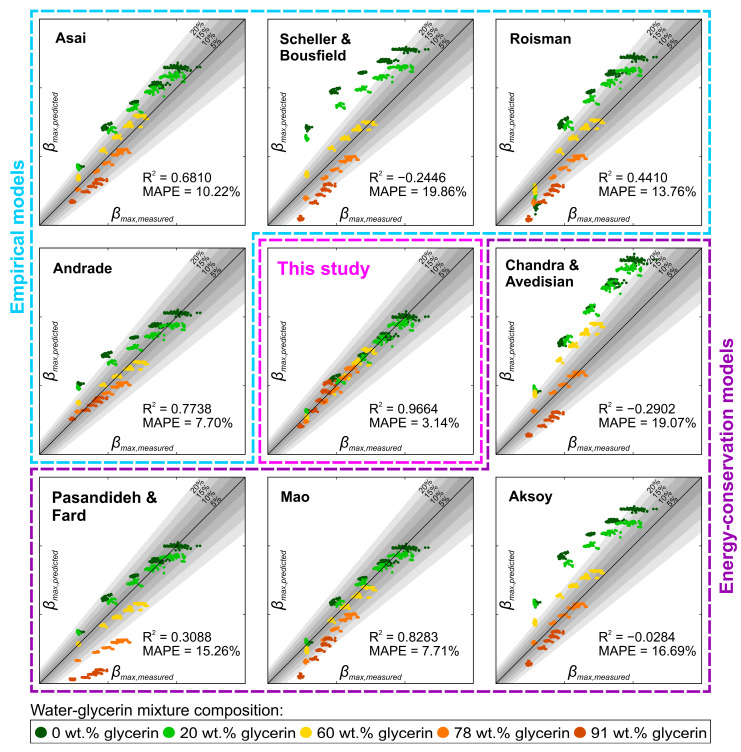
Comparison of correlations for predicting the maximum spreading coefficient, clearly highlighting the superiority of the current work [30,31,32,33,34,35,36,37]. Axis ranges in all charts correspond to values [1,4].

Interestingly, the observed scaling of *We* and *Re* with fractions of π potentially indicates that droplet geometry (e.g., volume) could be considered in the future when further developing this correlation. On the other hand, the value of the constant (i.e., 1.1885) represents the boundary case when the droplet is resting on the surface, radially deformed under the influence of gravity and surface energy. Once developed, we compare the accuracy of our empirical correlation for predicting the maximum spreading coefficient with other literature-derived models by calculating *β*_max_ using eight different empirical and energy conservation-based models, listed in Supplementary Material Table S5, and evaluating the deviation of their predictions from the measured values. The comparison between the model-predicted and experimentally measured maximum spreading coefficients shown in Figure 8 highlights the superior performance of our correlation, achieving an R^2^ value over 0.96 with a mean average percentage error of ~3%. Notably, our model maintains consistent agreement with the experimental data across different mixture compositions, whereas the literature-derived models, both empirical and energy conservation-based, tend to accurately predict the maximum spreading coefficient only at specific concentrations.

Rebound efficiency, on the other hand, displays an inverse trend with Weber and Reynolds numbers, with the latter also showcasing a distinct inflection point where the rebound appears to be the most effective energy-wise. The shape function during NLLS optimization, in this case, was constructed with three exponential functions with a natural base, yielding the following correlation after optimization:

(8) $$\eta =0.481\cdot e^{-\frac{1.8339}{100}We}\cdot \left(e^{-\frac{0.0934}{1000}Re}-e^{-\frac{4.1214}{1000}Re}\right)$$

Importantly, the trained IE GPR model for predicting *η* showcases a lower R^2^ value than the one used for *β*_max_ (see Table 1), which leads to less accurate data within the PDP-generated point cloud (*η*, *Re*, *We*). The latter leads to a relatively poor overall fit of the derived correlation (R^2^ values of 0.73), which mainly results from datapoints where low *Re* and *We* numbers are simultaneously present (i.e., when 15 ≤ *We* while 180 ≤ *Re*). If these points are excluded, our newly developed correlation predicts the rebound efficiency with noteworthy accuracy (R^2^ = 0.915), which could be further improved by additionally modeling the influence of the microchannel pitch, identified as the third most important parameter by the Shapley plot. Nevertheless, the current predictive performance within the augmented range far outperforms the few available models published in the literature [44,45,46]. Note here that both developed correlations are accurate for surfaces that exhibit the Cassie wetting state during droplet impact, whereas if the latter is absent or breaks down, the correlations are expected to produce less reliable estimates.

## 4. Conclusions

In this work, we present a novel use of data-driven isotropic exponential Gaussian process regression to model a large experimental dataset (1492 data vectors) of water–glycerin binary mixture droplet impacts on superhydrophobic aluminum. The trained and validated IE GPR model includes the most important droplet parameters (diameter and impact velocity) and surface topography (pitch and depth of laser-induced microchannels) while also containing fluid properties (density, dynamic viscosity and surface tension). From the analysis of individual parameter influence on three characteristic metrics—contact time, maximum spreading coefficient and rebound efficiency—we first confirm the dominant influence of droplet impact velocity and subsequently highlight that the droplet spreading phase appears independent of surface microtopography. Instead, during the retraction stage, the importance of unwetted area fraction is increased, denoted by higher rebound efficiency on samples with a smaller distance between laser-fabricated microchannels. Finally, the trained models are exploited to develop empirical correlations for predicting the maximum spreading coefficient and rebound efficiency, which strongly outperform all the currently published models, exhibiting goodness of fit of R^2^ = 0.966 and R^2^ = 0.915, respectively. The present work goes beyond the data-driven state of the art by covering all stages of droplet impact on functionalized surfaces, aiding future studies that aim to bridge the gap between the recorded macroscale surface–droplet interactions and the microscale properties of the interface or the thermophysical properties of the fluid.

## Figures and Tables

**Table 1:** Performance metrics of optimized IE GPR models for predictions with normalized *Re* and *We* numbers and surface topography.

Predicted Parameter	RMSE	MSE	R^2^	MAE
Rebound efficiency	0.017097	0.000292	0.9731	0.01051
Maximum spreading coefficient	0.037139	0.001379	0.9926	0.02082

## Data Availability

All data used within this study are available freely via a Mendeley Data repository at the following URL: https://data.mendeley.com/datasets/wsh8rxwd38/1 (accessed on 26 May 2025) and the following permanent DOI: dx.doi.org/10.17632/wsh8rxwd38.1.

## Supplementary Materials

The following supporting information can be downloaded at https://www.mdpi.com/article/10.3390/biomimetics10060357/s1, Table S1: Static and dynamic contact angles of water droplets and static contact angles of water–glycerin droplets with 91 wt.% glycerin for all tested samples; Table S2: Performance metrics with default model settings for predicting contact time with normalized data; Table S3: Performance metrics with default model settings for predicting the maximum spreading coefficient with normalized data; Table S4: Performance metrics with default model settings for predicting the rebound efficiency with normalized data; Table S5: List of literature-derived models for predicting *β*_max_.
