# Supplementary material for: Investigation of Droplet Spreading and Rebound Dynamics on Superhydrophobic Surfaces Using Machine Learning
Source: Biomimetics (Basel). 2025 Jun 1;10(6):357. doi: 10.3390/biomimetics10060357 (PMC12190483; doi:10.3390/biomimetics10060357)
Supplement: Supplementary file 1 [file biomimetics-10-00357-s001.zip › biomimetics-3644777-supplementary.pdf]

Supplementary Information

Investigation of Droplet Spreading and Rebound Dynamics on Superhydrophobic Surfaces Using Machine Learning

Samo Jereb, Jure Berce, Robert Lovšin, Matevž Zupančič, Matic Može* and Iztok Golobič

Faculty of Mechanical Engineering, University of Ljubljana, Aškerčeva cesta 6, SI-1000 Ljubljana, Slovenia

* *Correspondence: matic.moze@fs.uni-lj.si; Tel.: +386 1 4771 309*

Contents

Wettability of the samples 3
Selecting a machine learning model 4
List of literature-derived models for predicting β_{max} 8
Literature 9

Wettability of the samples

The wettability of the samples was assessed by measuring the static and dynamic contact angles of water droplets, with each measurement repeated five times. The average values of the static, advancing and receding contact angles, along with the resulting contact angle hysteresis, for all the samples featured in the dataset used to train the machine learning models are presented in Table S1. Additionally, we measured the static contact angle using a water-glycerin droplet with the highest content of glycerin (91 wt.%) featured in the rebound experiments to evaluate the potential influence of varying fluid's thermophysical properties, due to increased glycerin contact in the mixture, on surface wettability. The measured static contact angles of water-glycerin droplets are presented in the last column of Table S1. A comparison of the static contact angles of pure water and water-glycerin mixture shows that changes in mixture composition do not significantly affect the surface wettability, as the measured contact angles of both fluids for the same samples fall within the measurement uncertainty.

Table S1: Static and dynamic contact angles of water droplets and static contact angles of water-glycerin droplets with 91 wt.% glycerin for all tested samples.

Sample	Static contact angle [°]	Advancing contact angle [°]	Receding contact angle [°]	Contact angle hysteresis [°]	Water-glycerin static contact angle [°]
D50	163.1	162.4	156.9	5.5	160.8
D100	160.7	166.4	158.3	8.1	162.1
D200	162.2	165.3	158.4	6.9	164.0
D400	165.9	166.6	155.9	10.7	164.0
D600	160.8	165.4	158.5	6.8	160.0
D800	160.6	163.6	152.3	11.3	163.3
S50	165.2	165.1	159.5	5.7	164.2
S100	164.5	166.5	160.3	6.2	163.7
S200	163.2	165.3	159.7	5.6	162.8
S400	164.1	165.3	159.4	5.9	164.0
S600	163.2	165.9	158.9	7	162.6
S800	159.5	164.9	160.2	4.7	162.3

Selecting a machine learning model

The starting point for the presented study was the 28 models implemented in the Regression Learner Toolbox in MATHWORKS Matlab R2024b. The collection consists of distance-based and non-distance-based models, hence the data was first normalized using a standard Z-test normalization to remove the bias towards larger predictor values. Once the data was normalized, each model was trained with default settings on 80% of the data (while using 5-fold cross validation) and independently tested on the remaining 20%. This was done separately for each combination of predictions-predictor MISO model:

$$\{\tau_{\mathrm{c}}\,, \beta_{\mathrm{max}}, \eta\} = \boldsymbol{M}\big(D_{\mathrm{drop}}\,, v_{\mathrm{drop}}\,, \rho\,, \mu\,, \sigma\,, pitch\,, depth\big) \tag{S1}$$

effectively yielding the comparison of 28 model performances for individually predicting the contact time, rebound efficiency and spreading coefficient. The predictive performance parameters for each prediction output are given in Table S2 – Table S4, with colours indicating the best model according to each performance metric.

Table S2: Performance metrics with default model settings for predicting contact time with normalized data.

MODEL	VALIDATION				TESTING			
	RMSE	MSE	R^2	MAE	RMSE	MSE	R^2	MAE
Linear	0.00119	0.0000014	0.445	0.00091	0.00116	0.0000013	0.486	0.00088
Interactions Linear	0.00108	0.0000012	0.539	0.00080	0.00114	0.0000013	0.502	0.00080
Robust Linear	0.00121	0.0000015	0.425	0.00089	0.00120	0.0000014	0.451	0.00087
Stepwise Linear	0.00110	0.0000012	0.521	0.00083	0.00112	0.0000013	0.519	0.00082
Fine Tree	0.00109	0.0000012	0.529	0.00069	0.00112	0.0000013	0.517	0.00071
Medium Tree	0.00106	0.0000011	0.553	0.00070	0.00107	0.0000011	0.567	0.00070
Coarse Tree	0.00113	0.0000013	0.496	0.00080	0.00110	0.0000012	0.537	0.00075
Linear SVM	0.00150	0.0000022	0.117	0.00116	0.00139	0.0000019	0.264	0.00105
Quadratic SVM	0.00150	0.0000023	0.109	0.00115	0.00142	0.0000020	0.225	0.00106
Cubic SVM	0.00177	0.0000031	-0.237	0.00131	0.00155	0.0000024	0.080	0.00120
Fine Gaussian SVM	0.00128	0.0000016	0.357	0.00102	0.00132	0.0000017	0.334	0.00101
Medium Gaussian SVM	0.00135	0.0000018	0.275	0.00104	0.00134	0.0000018	0.310	0.00096
Coarse Gaussian SVM	0.00138	0.0000019	0.247	0.00099	0.00143	0.0000021	0.215	0.00099
Efficient Linear Least Squares	0.00118	0.0000014	0.446	0.00091	0.00116	0.0000013	0.486	0.00088
Efficient Linear SVM	0.00123	0.0000015	0.403	0.00089	0.00122	0.0000015	0.430	0.00088
Boosted Trees	0.00115	0.0000013	0.479	0.00081	0.00120	0.0000014	0.451	0.00083
Bagged Trees	0.00096	0.0000009	0.636	0.00063	0.00100	0.0000010	0.615	0.00064
Squared Exponential GPR	0.00097	0.0000009	0.630	0.00066	0.00107	0.0000011	0.562	0.00069
Matern 5/2 GPR	0.00095	0.0000009	0.647	0.00063	0.00107	0.0000011	0.565	0.00068
Exponential GPR	0.00092	0.0000008	0.665	0.00061	0.00106	0.0000011	0.572	0.00068
Rational Quadratic GPR	0.00093	0.0000009	0.660	0.00062	0.00106	0.0000011	0.568	0.00068
Narrow Neural Network	0.00122	0.0000015	0.412	0.00089	0.00113	0.0000013	0.510	0.00083
Medium Neural Network	0.00106	0.0000011	0.553	0.00077	0.00113	0.0000013	0.514	0.00081
Wide Neural Network	0.00232	0.0000054	-1.13	0.00140	0.00200	0.0000040	-0.53	0.00129
Bilayered Neural Network	0.00135	0.0000018	0.277	0.00095	0.00120	0.0000014	0.448	0.00092
Trilayered Neural Network	0.00114	0.0000013	0.486	0.00084	0.00122	0.0000015	0.427	0.00088
SVM Kernel	0.00107	0.0000011	0.550	0.00069	0.00113	0.0000013	0.510	0.00069
Least Squares Kernel Regression	0.00100	0.0000010	0.604	0.00069	0.00114	0.0000013	0.502	0.00077

*corresponding units of RMSE, MSE and MAE are seconds, while R^2 is dimensionless. The cell colours indicate model performance, with green representing the best and red the worst. The purple outline highlights the model that achieved the best overall performance.

Table S3: Performance metrics with default model settings for predicting the maximum spreading coefficient with normalized data.

MODEL	VALIDATION				TESTING			
	RMSE	MSE	R^2	MAE	RMSE	MSE	R^2	MAE
Linear	0.1481	0.02193	0.882	0.1137	0.1488	0.02213	0.884	0.1114
Interactions Linear	0.0456	0.00208	0.989	0.0326	0.0466	0.00217	0.989	0.0305
Robust Linear	0.1491	0.02222	0.880	0.1126	0.1504	0.02261	0.881	0.1108
Stepwise Linear	0.0462	0.00214	0.988	0.0330	0.0470	0.00221	0.988	0.0313
Fine Tree	0.0454	0.00206	0.989	0.0236	0.0398	0.00159	0.992	0.0206
Medium Tree	0.0499	0.00249	0.987	0.0284	0.0383	0.00147	0.992	0.0228
Coarse Tree	0.0799	0.00638	0.966	0.0538	0.0626	0.00391	0.979	0.0394
Linear SVM	0.1503	0.02260	0.878	0.1136	0.1531	0.02343	0.877	0.1126
Quadratic SVM	0.0465	0.00216	0.988	0.0349	0.0476	0.00227	0.988	0.0337
Cubic SVM	0.0378	0.00143	0.992	0.0284	0.0397	0.00158	0.992	0.0290
Fine Gaussian SVM	0.0616	0.00380	0.980	0.0453	0.0536	0.00287	0.985	0.0407
Medium Gaussian SVM	0.0404	0.00163	0.991	0.0301	0.0400	0.00160	0.992	0.0288
Coarse Gaussian SVM	0.0671	0.00450	0.976	0.0449	0.0588	0.00346	0.982	0.0396
Efficient Linear Least Squares	0.1481	0.02194	0.882	0.1140	0.1487	0.02211	0.884	0.1116
Efficient Linear SVM	0.1504	0.02263	0.878	0.1137	0.1531	0.02345	0.877	0.1126
Boosted Trees	0.0999	0.00997	0.946	0.0886	0.0990	0.00981	0.948	0.0861
Bagged Trees	0.0378	0.00143	0.992	0.0221	0.0395	0.00156	0.992	0.0220
Squared Exponential GPR	0.0307	0.00094	0.995	0.0200	0.0321	0.00103	0.995	0.0189
Matern 5/2 GPR	0.0292	0.00085	0.995	0.0185	0.0306	0.00094	0.995	0.0182
Exponential GPR	0.0289	0.00084	0.995	0.0168	0.0270	0.00073	0.996	0.0156
Rational Quadratic GPR	0.0297	0.00088	0.995	0.0190	0.0313	0.00098	0.995	0.0187
Narrow Neural Network	0.0364	0.00133	0.993	0.0243	0.0383	0.00147	0.992	0.0241
Medium Neural Network	0.0322	0.00104	0.994	0.0216	0.0343	0.00118	0.994	0.0209
Wide Neural Network	0.0297	0.00088	0.995	0.0180	0.0317	0.00101	0.995	0.0182
Bilayered Neural Network	0.0326	0.00106	0.994	0.0213	0.0339	0.00115	0.994	0.0210
Trilayered Neural Network	0.0330	0.00109	0.994	0.0208	0.0326	0.00106	0.994	0.0191
SVM Kernel	0.0420	0.00177	0.990	0.0322	0.0402	0.00161	0.992	0.0286
Least Squares Kernel Regression	0.0662	0.00439	0.976	0.0474	0.0597	0.00356	0.981	0.0449

*all parameters (RMSE, MSE, MAE and R^2) are dimensionless. The cell colours indicate model performance, with green representing the best and red the worst. The purple outline highlights the model that achieved the best overall performance.

Table S4: Performance metrics with default model settings for predicting the rebound efficiency with normalized data.

**MODEL**	**VALIDATION**				**TESTING**			
	RMSE	MSE	R^2	MAE	RMSE	MSE	R^2	MAE
Linear	0.03960	0.001568	0.856	0.03121	0.04046	0.001637	0.847	0.03122
Interactions Linear	0.02644	0.000699	0.936	0.02071	0.02873	0.000825	0.923	0.02202
Robust Linear	0.04015	0.001612	0.852	0.03044	0.04095	0.001677	0.843	0.03058
Stepwise Linear	0.02637	0.000695	0.936	0.02059	0.02846	0.000810	0.924	0.02192
Fine Tree	0.01781	0.000317	0.971	0.01074	0.01788	0.000320	0.970	0.01005
Medium Tree	0.02129	0.000453	0.958	0.01354	0.02498	0.000624	0.942	0.01416
Coarse Tree	0.02640	0.000697	0.936	0.01768	0.02512	0.000631	0.941	0.01618
Linear SVM	0.04079	0.001664	0.847	0.03027	0.04166	0.001736	0.838	0.03066
Quadratic SVM	0.01890	0.000357	0.967	0.01251	0.02158	0.000466	0.956	0.01431
Cubic SVM	0.01612	0.000260	0.976	0.01063	0.01652	0.000273	0.974	0.01049
Fine Gaussian SVM	0.02063	0.000426	0.961	0.01256	0.02190	0.000480	0.955	0.01305
Medium Gaussian SVM	0.01690	0.000286	0.974	0.01161	0.01840	0.000339	0.968	0.01228
Coarse Gaussian SVM	0.03010	0.000906	0.917	0.02239	0.03047	0.000928	0.913	0.02320
Efficient Linear Least Squares	0.03960	0.001569	0.856	0.03119	0.04053	0.001642	0.846	0.03124
Efficient Linear SVM	0.04073	0.001659	0.847	0.03028	0.04172	0.001741	0.837	0.03066
Boosted Trees	0.01867	0.000349	0.968	0.01354	0.02014	0.000405	0.962	0.01381
Bagged Trees	0.01812	0.000328	0.970	0.01077	0.01993	0.000397	0.963	0.01119
Squared Exponential GPR	0.01547	0.000239	0.978	0.00962	0.01640	0.000269	0.975	0.00992
Matern 5/2 GPR	0.01506	0.000227	0.979	0.00909	0.01653	0.000273	0.974	0.00974
Exponential GPR	0.01456	0.000212	0.981	0.00834	0.01711	0.000293	0.973	0.00958
Rational Quadratic GPR	0.01509	0.000228	0.979	0.00903	0.01669	0.000279	0.974	0.00974
Narrow Neural Network	0.01699	0.000289	0.973	0.01126	0.01965	0.000386	0.964	0.01312
Medium Neural Network	0.01619	0.000262	0.976	0.01071	0.01634	0.000267	0.975	0.01044
Wide Neural Network	0.01619	0.000262	0.976	0.00940	0.02044	0.000418	0.961	0.01074
Bilayered Neural Network	0.01670	0.000279	0.974	0.01017	0.01726	0.000298	0.972	0.01063
Trilayered Neural Network	0.01668	0.000278	0.974	0.01046	0.01980	0.000392	0.963	0.01209
SVM Kernel	0.01708	0.000292	0.973	0.01125	0.01926	0.000371	0.965	0.01172
Least Squares Kernel Regression	0.02058	0.000423	0.961	0.01478	0.02100	0.000441	0.959	0.01528

*all parameters (RMSE, MSE, MAE and R^2) are dimensionless. The cell colours indicate model performance, with green representing the best and red the worst. The purple outline highlights the model that achieved the best overall performance.

List of literature-derived models for predicting β_{max}

Table S5 lists eight different literature-derived models for predicting the maximum spreading coefficient of a droplet impacting on a solid surface, which were used to evaluate the accuracy of our correlation. The models are categorized into two groups based on their derivation: empirical scaling of dimensionless numbers and physical modeling based on energy conservation.

Table S5: List of literature-derived models for predicting β_{max}

Authors	Empirical-scaling models
Asai et al. [30]	$\beta_{\mathrm{max}} = 1 + 0.48\ \mathrm{We}^{0.5} \times \exp[-1.48\ \mathrm{We}^{0.22}\mathrm{Re}^{-0.21}]$
Scheller & Bousfield [31]	$\beta_{\mathrm{max}} = 0.61\ (\mathrm{Re}^2 \cdot \mathrm{Oh})^{0.166}$
Roisman [32]	$\beta_{\mathrm{max}} = 0.87\ \mathrm{Re}^{0.2} - 0.4\ \mathrm{Re}^{0.4}/\sqrt{\mathrm{We}}$
Andrade et al. [33]	$\beta_{\mathrm{max}} = 1.28 + 0.071\ \mathrm{We}^{1/4}\ \mathrm{Re}^{1/4}$
Authors	Energy-conservation-based models
Chandra & Avedisian [34]	$1.5\ \mathrm{We}/\mathrm{Re}\ \beta_{\mathrm{max}}^4 + (1-\cos\theta)\beta_{\mathrm{max}}^2 - (1/3\ \mathrm{We} + 4) \approx 0$
Pasandideh-Fard et al. [35]	$\beta_{\mathrm{max}} = \sqrt{\frac{\mathrm{We}+12}{3\ (1-\cos\theta) + 4\ \mathrm{We}/\sqrt{\mathrm{Re}}}}$
Mao et al. [36]	$\left[\frac{1}{4}(1-\cos\theta) + 0.2\frac{\mathrm{We}^{0.83}}{\mathrm{Re}^{0.33}}\right]\beta_{\mathrm{max}}^3 - \left(\frac{\mathrm{We}}{12}+1\right)\beta_{\mathrm{max}} + \frac{2}{3} = 0$
Aksoy et al. [37]	$3.18\frac{\mathrm{We}^{0.72}}{\mathrm{Re}^{0.86}}\beta_{\mathrm{max}}^{6.5} + 3(1-\cos\theta)\beta_{\mathrm{max}}^3 - (\mathrm{We}+12)\beta_{\mathrm{max}} + 8 = 0$

Literature

30. Asai, A.; Shioya, M.; Hirasawa, S.; Okazaki, T. Impact of an Ink Drop on Paper. *207*, 205–207.

31. Scheller, B.L.; Bousfield, D.W. Newtonian Drop Impact with a Solid Surface. AIChE J. 1995, 41, 1357–1367, doi:10.1002/aic.690410602.

32. Roisman, I. V. Inertia Dominated Drop Collisions. II. An Analytical Solution of the Navier-Stokes Equations for a Spreading Viscous Film. Phys. Fluids 2009, 21, doi:10.1063/1.3129283.

33. Andrade, R.; Skurtys, O.; Osorio, F. Experimental Study of Drop Impacts and Spreading on Epicarps : Effect of Fluid Properties. J. Food Eng. 2012, 109, 430–437, doi:10.1016/j.jfoodeng.2011.10.038.

34. Chandra, S.; Avedisian, C.T. On the Collision of a Droplet with a Solid Surface. Proc. R. Soc. A Math. Phys. Eng. Sci. 1991, 432, 13–41, doi:10.1098/rspa.1991.0002.

35. Pasandideh-Fard, M.; Qiao, Y.M.; Chandra, S.; Mostaghimi, J. Capillary Effects during Droplet Impact on a Solid Surface. Phys. Fluids 1996, 8, 650–659, doi:10.1063/1.868850.

36. Mao, T.; Kuhn, D.C.S.; Tran, H. Spread and Rebound of Liquid Droplets upon Impact on Flat Surfaces. 1997, 4, 2169–2179.

37. Aksoy, Y.T.; Eneren, P.; Koos, E.; Vetrano, M.R. Spreading of a Droplet Impacting on a Smooth Flat Surface: How Liquid Viscosity Influences the Maximum Spreading Time and Spreading Ratio. Phys. Fluids 2022, 34, doi:10.1063/5.0086050.
